# Exacerbated Airway Toxicity of Environmental Oxidant Ozone in Mice Deficient in *Nrf2*

**DOI:** 10.1155/2013/254069

**Published:** 2013-05-09

**Authors:** Hye-Youn Cho, Wesley Gladwell, Masayuki Yamamoto, Steven R. Kleeberger

**Affiliations:** ^1^Laboratory of Respiratory Biology, National Institute of Environmental Health Sciences, National Institutes of Health, Research Triangle Park, NC 27709, USA; ^2^Tohoku University Graduate School of Medicine, Sendai 980-8575, Japan

## Abstract

Ozone (O_3_) is a strong oxidant in air pollution that has harmful effects on airways and exacerbates respiratory disorders. The transcription factor Nrf2 protects airways from oxidative stress through antioxidant response element-bearing defense gene induction. The present study was designed to determine the role of Nrf2 in airway toxicity caused by inhaled O_3_ in mice. For this purpose, *Nrf2*-deficient (*Nrf*2^−/−^) and wild-type (*Nrf*2^+/+^) mice received acute and subacute exposures to O_3_. Lung injury was determined by bronchoalveolar lavage and histopathologic analyses. Oxidation markers and mucus hypersecretion were determined by ELISA, and Nrf2 and its downstream effectors were determined by RT-PCR and/or Western blotting. Acute and sub-acute O_3_ exposures heightened pulmonary inflammation, edema, and cell death more severely in *Nrf*2^−/−^ mice than in *Nrf*2^+/+^ mice. O_3_ caused bronchiolar and terminal bronchiolar proliferation in both genotypes of mice, while the intensity of compensatory epithelial proliferation, bronchial mucous cell hyperplasia, and mucus hypersecretion was greater in *Nrf*2^−/−^ mice than in *Nrf*2^+/+^ mice. Relative to *Nrf*2^+/+^, O_3_ augmented lung protein and lipid oxidation more highly in *Nrf*2^−/−^ mice. Results suggest that *Nrf2* deficiency exacerbates oxidative stress and airway injury caused by the environmental pollutant O_3_.

## 1. Introduction

Ozone (O_3_) is a highly reactive gaseous oxidant air pollutant. Elevated levels of ambient O_3_ have been associated with increased hospital visits and respiratory symptoms including chest discomfort, breathing difficulties, coughing, and lung function decrement [1, 2]. Moreover, subjects with preexisting asthma and rhinitis are known to be particularly vulnerable to O_3_ and are at risk of exacerbations [3]. Controlled O_3_ exposure studies in healthy volunteers found oxidant generation and temporal antioxidant depletion in fluid lining compartments of the airways or sputum [4]. Inhaled O_3_ in experimental animal models causes airway inflammation and hyperresponsiveness, reactive oxygen species (ROS) production, mucus overproduction, and epithelial damage and compensatory proliferation predominantly in ciliated cells of the upper respiratory tract and Clara cells in terminal bronchioles. Long-term exposure of O_3_ may cause lung tumors in certain strains of mice [5].

Many studies have investigated the roles of inflammatory mediators in the pathogenic airway response to O_3_. Infiltration of neutrophils into the interstitium and airways contributes to O_3_-induced nasal mucous cell metaplasia and airway hyperreactivity [6, 7], although some studies demonstrated uncoupling of airway inflammation and hyperreactivity [8, 9]. Tumor-necrosis-factor- (TNF-) *α*, a susceptibility gene for O_3_ toxicity in mice [10], has a significant role in O_3_-induced inflammation and airway hyperreactivity in rodent lungs mediated through nuclear factor-*κ*B and activator protein-1 [10–13]. Toll-like receptor 4 and inflammasome proteins (e.g., Nlrp3) also contribute to O_3_-induced airway hyperpermeability and hyperreactivity, respectively, in mice [14–16]. 

O_3_ is thought to initiate toxicity by oxidation of biomolecules including proteins and lipids in epithelial lining fluid (ELF) of the airways, which is believed to activate signaling cascades and initiate inflammatory sequelae [17]. Nonenzymatic antioxidants in the ELF that protect membranes and macromolecules include uric acid, ascorbic acid, tocopherol, and glutathione (GSH), and their protective roles against O_3_ have been investigated chemically [18] and biologically [19, 20]. Enzymatic antioxidant and defense proteins bearing *cis*-acting antioxidant response elements (AREs) for the transcription factor nuclear NF-E2-related factor 2 (Nfe2l2, Nrf2) binding are particularly abundant in cellular and extracellular compartments of airway tissues. It has been determined that O_3_ causes increases of ARE-responsive antioxidants including direct, scavenging enzymes (e.g., superoxide dismutases (SODs)) and indirect, defense enzymes (e.g., glutathione-S-transferase (GST), heme oxygenase-1 (HO-1)) in the lung [17, 21]. More recent studies indicated that O_3_ increased pulmonary Nrf2 *in vivo* or *in vitro* [22–24]. Protective roles of Nrf2 and ARE-responsive antioxidant effectors against O_3_ toxicity are thus implicit while their functions are not well understood.

 The current study was designed to test the hypothesis that Nrf2 protects the lung against the pathogenesis of O_3_-induced injury in the mouse. For this purpose, mice deficient in *Nrf2* (*Nrf*2^−/−^) and their wild-type controls (*Nrf*2^+/+^) were exposed to O_3_ using two models. Acute exposure (3 hr) to 2 parts per million (ppm) O_3_ caused airway inflammation characterized by neutrophil inflammation that peaks approximately 6 hr after exposure and induced airways hyperreactivity approximately 24 hr after exposure. Subacute exposure (24–72 hr) to 0.3 ppm O_3_ caused airways inflammation. Use of both exposure models enabled us to evaluate the role of Nrf2 for multiple O_3_-related phenotypes by comparing responses between two genotypes.

## 2. Materials and Methods

### 2.1. Mice

Breeding colonies of *Nrf*2^+/+^ and *Nrf*2^−/−^ mice [25] were backcrossed to ICR (Taconic, Hudson, NY, USA) as previously published [26] and maintained in the National Institute of Environmental Health Sciences (NIEHS) animal facility. Mice were provided with modified AIN-76A diet and water *ad libitum*. 

### 2.2. Inhalation Exposure

After acclimation, mice were placed in individual stainless-steel wire cages within a whole-body inhalation chamber (Hazelton 1000; Lab Products, Maywood, NJ, USA) equipped with a charcoal and high-efficiency particulate air-filtered air supply. Mice had free access to water and food. For the sub-acute model, mice were exposed continuously to 0.3 ppm O_3_ for 6, 24, 48, or 72 hr. For the acute model, mice were exposed continuously to 2 ppm O_3_ for 3 hr and recovered in room air for 3, 6, or 24 hr. O_3_ was generated from ultrahigh purity air (<1 ppm total hydrocarbons; National Welders Inc., Raleigh, NC, USA) using a silent arc discharge O_3_ generator (Model L-11, Pacific Ozone Technology, Benicia, CA, USA). Constant chamber air temperature (72 ± 3°F) and relative humidity (50 ± 15%) were maintained. O_3_ concentration was monitored continually (Dasibi model 1008-PC, Dasibi Environmental Corp.). Parallel exposure to filtered air was done in a separate chamber for the same duration. Immediately following each exposure, mice were euthanized by sodium pentobarbital overdose (104 mg/Kg). All animal use was approved by the NIEHS Animal Care and Use Committee. 

### 2.3. Measurement of Airways Reactivity

At the end of designated exposure duration, mice were anesthetized with urethane (1.5 g/kg in 0.125 *μ*g/*μ*L PBS, *i.p.*), placed on a temperature controlled heating pad, and connected to an EKG monitor. A tracheal cannula was surgically inserted and attached to a small animal ventilator equipped with a nebulizer. After loss of responses to pain stimulus (foot pinch), mice were paralyzed with pancuronium bromide injection (0.8 mg/kg as 0.08 mg/mL PBS) and subjected to a deep lung inflation. Lung function was measured using a computer controlled flow-type body plethysmograph system (FlexiVent; SciReq Inc., Montreal, QC, Canada). Mice were ventilated at a respiratory rate of 150 breaths/min and tidal volume of 10 mL/kg against a positive end expiratory pressure of 3 cm H_2_O. Following baseline resistance measurements, mice were challenged with increasing doses of acetylcholine aerosol (6.25, 12.5, or 25 mg/mL). Lung function parameters were acquired by fitting pressure and volume data to the single compartment model and the constant-phase model measuring parameters including resistance of the whole respiratory system as described by the manufacturer. From the plot of resistance against acetylcholine concentration, area under the curve (AUC) of resistance was calculated. 

### 2.4. Bronchoalveolar Lavage (BAL) Analyses

Right lungs from each mouse were lavaged* in situ* with HBSS, and BAL returns were analyzed for total protein content and cell differentials as described previously [11]. 

### 2.5. Lung Histopathology

Left lung tissues from each mouse were inflated gently with 10% neutrally buffered formalin, fixed under constant pressure for 30 min, and proximal (around generation 5) and distal (approximately generation 11) levels of the main axial airway were sectioned for paraffin embedding. Tissue sections (5 *μ*m thick) were stained with H&E and AB/PAS.

### 2.6. Sandwich Enzyme-Linked Immunosorbent Assay (ELISA) of Mucin

Secreted mucin 5, subtypes A and C (Muc5AC) protein was determined with adaptation of a published method [27, 28]. Briefly, an aliquot of BAL fluid (20 *μ*L) was loaded in each well of an ELISA plate containing a polyclonal anti-Muc5AC capture antibody (1 : 40 dilution; sc-19603, Santa Cruz Biotechnology Inc., Santa Cruz, CA, USA) in pH 9.5 bicarbonate-carbonate coating buffer (BD OptEIA Reagent; BD Biosciences Pharmingen, San Diego, CA, USA). The plate was incubated at 48°C until the reaction was dry (>5 hr). The wells were washed and blocked overnight with an assay diluent containing 10% fetal bovine serum (BD Opt EIA) at 4°C. The samples were then incubated with a 1 : 100 diluted biotinylated monoclonal anti-Muc5AC detection antibody (Clone 45M1; Thermo Scientific/Lab Vision Co., Fremont, CA, USA) for 1.5 hr at 37°C. Following incubation with a peroxidase-conjugated secondary antibody (1 : 2500, goat anti-mouse-IgG-HRP), color change was developed by adding the TMB substrate solution. Optical density was measured at 450 nm after the stop buffer was added.

### 2.7. Redox Measurement

The amount of oxidized protein was quantified in lung protein aliquots by colorimetric detection of protein carbonyls [29]. Briefly, total lung protein samples (1 *μ*g) were adsorbed onto a 96-well plate (OxiSelect Protein Carbonyl ELISA; Cell Biolabs Inc., San Diego, CA, USA) overnight at 4°C. After derivatization of the protein carbonyls moieties by adding 2,4-dinitrophenylhydrazine (DNP), the protein samples were incubated with an anti-DNP antibody and a secondary antibody in turn following the manufacturer's instructions. The protein carbonyl contents were quantified by absorbance at 450 nm using a standard curve from predetermined reduced and oxidized BSA standards. Lung lipid oxidation was determined by measuring the amount of malondialdehyde (MDA) which forms 1 : 2 adduct with thiobarbituric acid (TBA). Briefly, an aliquot of lung homogenates (equivalent to 50 *μ*g proteins) was incubated with TBA reactive substances (OxiSelect TBARS Assay; Cell Biolabs Inc.) at 95°C for 1 hr. Color change indicating MDA-TBA adducts was measured spectrophotometrically at 532 nm, and MDA was quantified using a standard curve. Total glutathione levels in airway ELF were quantified by a kinetic method in an aliquot of BAL fluid (20 *μ*L) following the manufacturer's instruction (OxiSelect Total Glutathione Assay; Cell Biolabs Inc.). Briefly, oxidized glutathione (GSSG) in the sample was reduced to GSH by adding glutathione reductase in the presence of NADPH and subsequently adding chromogen for reaction with the thiol group of GSH, which produced a colored compound that was detectable at 405 nm. Total GSH concentration proportional to the rate of chromophore production was determined by comparison with the predetermined GSH standard curve.

### 2.8. RT-PCR

cDNA was prepared from total lung RNA of each mouse (*n* = 3-4/group), and quantitative PCR was performed following a published procedure [30] using 240 nM of primer sets specific for glutathione peroxidase 2 ((GPx2) 381 forward 5′-tgc aac cag ttc gga cat c-3′, 531 reverse 5′-agg caa aga cag gat gct c-3′), HO-1 (901 forward 5′-aga tca gca cta gct cat ccc-3′, 1074 reverse 5′-gcc agg caa gat tct ccc tta-3′), or NADP(H):quinone oxidoreductase 1 ((NQO1) 1141 forward 5′-agc gag ctg gaa aat act ct-3′, 1303 reverse 5′-ggc cat tgt tta ctt tga gc-3′) in a 7700 prism sequence detection system (Applied Biosystems, Carlsbad, CA, USA). Semiquantitative PCR was done for Nrf2 message [29]. 

### 2.9. Western Blot Analysis

Lung total proteins (50 *μ*g) isolated from RIPA homogenates were separated on appropriate percentage Tris-HCl SDS-PAGE gels (Bio-Rad Laboratories, Hercules, CA, USA) and analyzed by routine Western blotting using specific antibodies against Nrf2 (Santa Cruz Biotechnology Inc.) and pan-actin (Santa Cruz Biotechnology Inc.). Representative protein blot images from duplicates were scanned using the Bio-Rad Gel Doc system.

### 2.10. Statistics

 SigmaPlot 11.0 (Systat Software Inc., San Jose, CA, USA) was used to compare means. One-way ANOVA followed by Student-Newman-Keuls test for *a posteriori* comparisons was used for Nrf2 mRNA data sets. Two-way ANOVA followed by Student-Newman-Keuls test was used for other data sets. Data were expressed as group mean ± SEM. A *P* value less than 0.05 was considered statistically significant.

## 3. Results

### 3.1. Lung Injury Parameters in BAL

Overall, compared to acute O_3_ exposure, sub-acute O_3_ exposure caused greater pulmonary protein edema determined by total protein concentration and airway cell lysis determined by lactate dehydrogenase level by 72 hr exposure. In contrast, acute O_3_ exposure caused more pronounced inflammatory cell influx to the airways than sub-acute exposures. The degree of airway epithelial cell exfoliation was similar in both models.

#### 3.1.1. Sub-Acute *O*_3_

**Figure 1:**
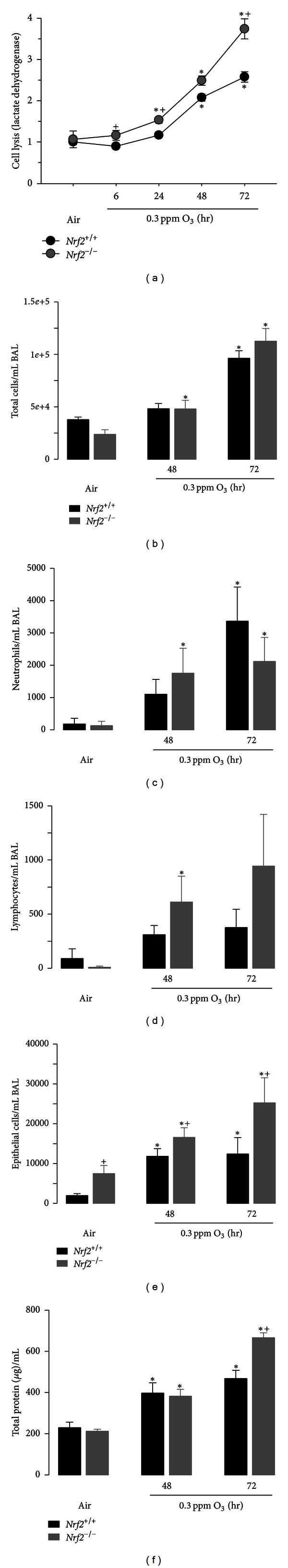
Lung injury after sub-acute O_3_ exposure. Lactate dehydrogenase levels (a), total cells (b), neutrophils (c), lymphocytes (d), epithelial cells (e), and total protein concentrations (f) in fluid recovered by bronchoalveolar lavage (BAL) from *Nrf*2^+/+^ and *Nrf*2^−/−^ mice after 6, 24, 48, or 72 hr exposure to 0.3 ppm O_3_. Control mice were exposed to filtered air. All data are presented as mean ± SEM. *Significantly different from genotype-matched air controls (*P* < 0.05). ^+^Significantly different from exposure-matched *Nrf*2^+/+^ mice (*P* < 0.05). *n* = 5 (air) or 12 (O_3_) per group.

With the exception of epithelial cells, no significant differences in the mean number of cellular phenotypes were found between *Nrf*2^−/−^ and *Nrf*2^+/+^ mice after air exposure. However, 0.3 ppm O_3_ caused significant lung edema, cellular injury, and inflammatory cell influx in both genotypes of mice, which were maximal after 72 hr exposure (Figure 1). Relative to *Nrf*2^+/+^ mice, significantly heightened lung cell cytotoxicity indicated by BAL lactate dehydrogenase level, edema indicated by total BAL protein concentration, and epithelial exfoliation were found in *Nrf*2^−/−^ mice (Figure 1). However, no significant difference was observed in mean numbers of BAL neutrophils between the genotypes after O_3_ (Figure 1). 

#### 3.1.2. Acute *O*_3_

**Figure 2:**
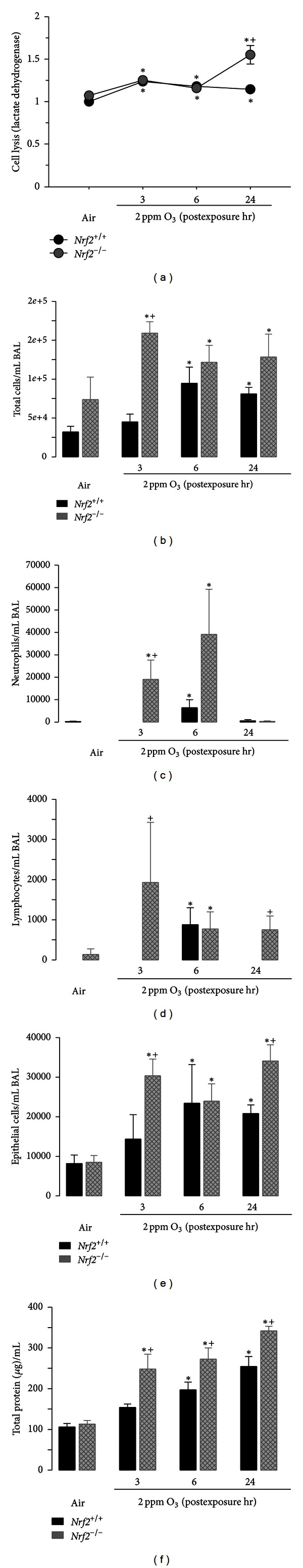
Lung injury after acute O_3_ exposure. Lactate dehydrogenase levels (a), total cells (b), neutrophils (c), lymphocytes (d), epithelial cells (e), and total protein concentrations (f) in fluid recovered by bronchoalveolar lavage (BAL) from *Nrf*2^+/+^ and *Nrf*2^−/−^ mice 3, 6, or 24 hr after 3 hr exposure to 2 ppm O_3_. Control mice were exposed to filtered air. All data are presented as mean ± SEM. *Significantly different from genotype-matched air controls (*P* < 0.05). ^+^Significantly different from exposure-matched *Nrf*2^+/+^ mice (*P* < 0.05). *n* = 5–8 per group.

No significant differences in mean BAL phenotypes were found between *Nrf*2^−/−^ and *Nrf*2^+/+^ mice after air exposure. Relative to sub-acute O_3_ exposure that caused mild-to-moderate BAL phenotype changes, 2 ppm O_3_ caused acute phase inflammatory responses characterized by neutrophilic influx (Figure 2). Significantly greater mean numbers of BAL neutrophils, epithelial cells, and total protein concentration were found as early as 3 hr postexposure (PE) in *Nrf*2^−/−^ mice compared to *Nrf*2^+/+^ mice (Figure 2). BAL cell lysis was also significantly greater in *Nrf*2^−/−^ mice than in *Nrf*2^+/+^ mice at 24 hr PE (Figure 2). 

### 3.2. Airway Reactivity

Total airway response to acetylcholine indicated by AUC was measured at 24 hr PE after 2 ppm O_3_ exposure. Mice exposed to either air or O_3_ did not respond differently to aerosolized acetylcholine compared to vehicle (see Supplementary Figure 1 available online at http://dx.doi.org/10.1155/2013/254069). Although dose response pattern to acetylcholine was observed in AUC regardless of the genotype and exposure, genetic deletion of *Nrf2* did not significantly alter airway responsiveness basally or after O_3_ (Supplementary Figure 1).

### 3.3. Pulmonary Histopathology

**Figure 3:**
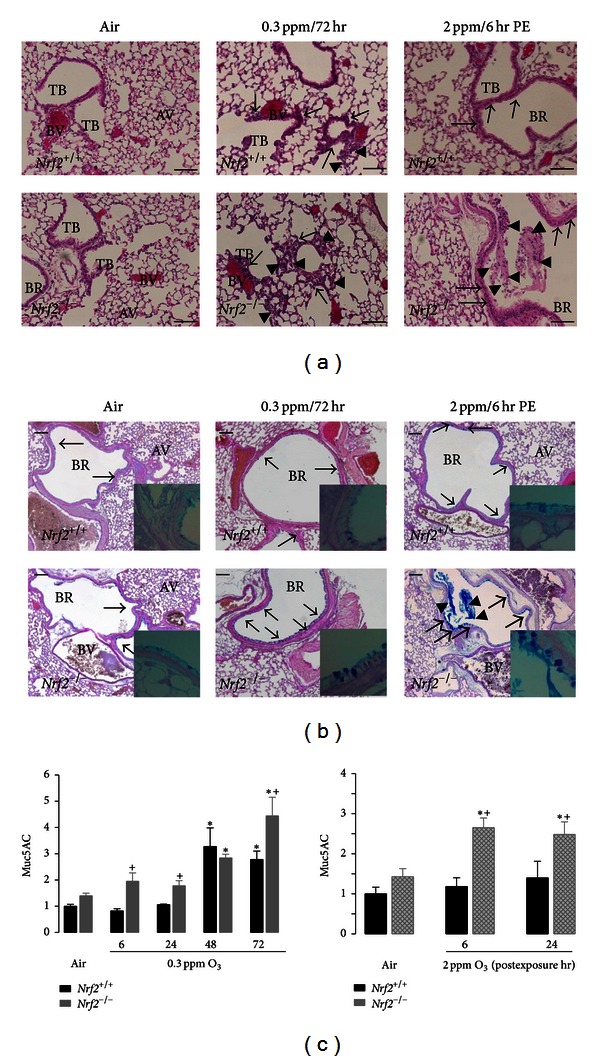
Lung histopathology and mucus hypersecretion. (a) Epithelial proliferation lining terminal bronchioles and alveoli accompanying air space infiltration of inflammatory cells in *Nrf*2^+/+^ (top panels) and *Nrf*2^−/−^ (bottom panels) mice after air (left panels), 72 hr exposure to 0.3 ppm O_3_ (middle panels), and 6 hr postexposure to 2 ppm O_3_ (right panels). Representative light photomicrographs of H&E-stained lung tissue sections are presented. Arrows indicate proliferation of epithelial cells. Arrow heads indicate infiltrated inflammatory cells. AV: alveoli; BR: bronchi or bronchiole; TB: terminal bronchiole; BV: blood vessel. Bars = 100 *μ*m. (b) AB/PAS-positive mucous goblet cells in *Nrf*2^+/+^ (top panels) and *Nrf*2^−/−^ (bottom panels) mice after air (left panels), 72 hr exposure to 0.3 ppm O_3_ (middle panels), and 6 hr post-exposure to 2 ppm O_3_ (right panels). Inlets are higher magnification of mucus stored in bronchial epithelial goblet cells. Representative light photomicrographs of AB/PAS-stained lung tissue sections are presented. Arrows indicate intraepithelial mucosubstances. Arrow heads indicate secreted mucus in air space. Bars = 100 *μ*m. (c) Amount of Muc5AC proteins in secreted mucus determined by ELISA in BAL returns from *Nrf*2^+/+^ and *Nrf*2^−/−^ mice after air or O_3_ exposure. All data are presented as mean ± SEM (*n* = 3-4/group). *Significantly different from genotype-matched air controls (*P* < 0.05). ^+^Significantly different from exposure-matched *Nrf*2^+/+^ mice (*P* < 0.05).

Compared to air exposure, 0.3 ppm O_3_ caused mild histologic changes in *Nrf*2^+/+^ lungs characterized by thickening of epithelium lining bronchioles and terminal bronchioles indicating epithelial cell proliferation and by neutrophil influx in air spaces after 72 hr (Figure 3(a)). More severe proliferation was found in *Nrf*2^−/−^ mice exposed to 0.3 ppm O_3_, which extended to alveolar epithelium in addition to terminal bronchial epithelium and coincided with inflammatory cell accumulation (Figure 3(a)). Consistent with the BAL phenotypes, 2 ppm O_3_ caused histologically evident inflammatory cell influx to the air spaces particularly in *Nrf*2^−/−^ mice from 6 hr PE (Figure 3(a)). The abundance of AB/PAS-positive mucus-bearing goblet cells in main stem airway epithelium was increased in both genotypes after 0.3 ppm O_3_, while this mucous cell hyperplasia was more manifest in *Nrf*2^−/−^ mice than in *Nrf*2^+/+^ mice (Figure 3(b)). Acute O_3_ also caused bronchial mucous cell hyperplasia and airway mucus hypersecretion more noticeably in *Nrf*2^−/−^ mice than in *Nrf*2^+/+^ mice (Figure 3(b)). As assessed by Muc5AC protein amounts in BAL fluids, mucus hypersecretion was found earlier and/or in greater amounts in *Nrf*2^−/−^ mice compared to *Nrf*2^+/+^ mice after sub-acute and acute exposures (Figure 3(c)). 

### 3.4. Pulmonary Redox Status

**Figure 4:**
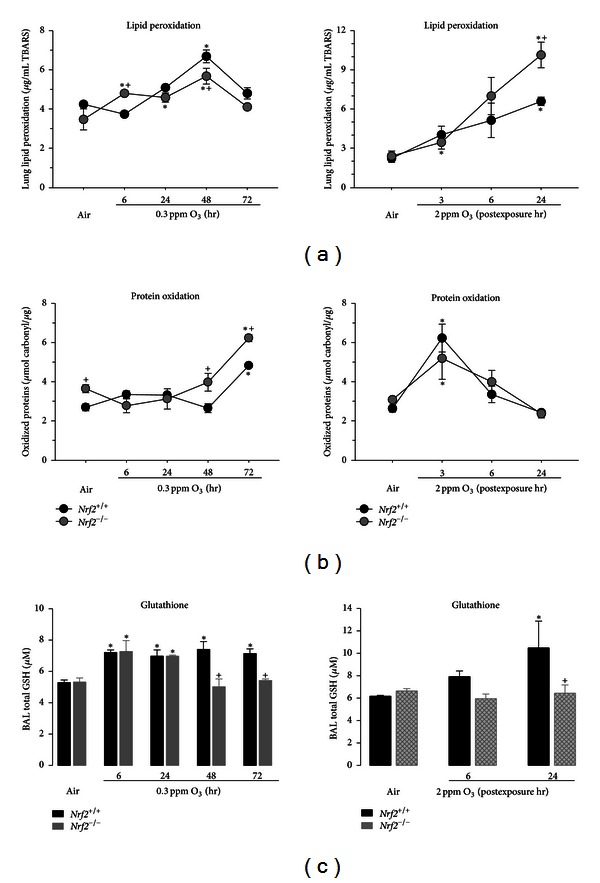
Lung redox status. (a) Malondialdehyde (MDA) levels conjugated with the substrate TBARS in lung homogenates from *Nrf*2^+/+^ and *Nrf*2^−/−^ mice after 6 hr or 24, 48, and 72 hr exposure to 0.3 ppm O_3_ (left) and 3, 6, and 24 hr after 3 hr exposure to 2 ppm O_3_ (right). *n* = 3/group. (b) Oxidized protein levels in lung homogenates from *Nrf*2^+/+^ and *Nrf*2^−/−^ mice after exposure to air, 0.3 ppm O_3_ (left), or 2 ppm O_3_ (right). *n* = 3/group. (c) Total glutathione (GSH) in bronchoalveolar lavage returns (100 *μ*L) from *Nrf*2^+/+^ and *Nrf*2^−/−^ mice after exposure to air, 0.3 ppm O_3_ (left), or 2 ppm O_3_ (right). *n* = 3/group. All data are presented as mean ± SEM. *Significantly different from genotype-matched air control mice (*P* < 0.05). ^+^Significantly lower than exposure-matched *Nrf*2^+/+^ mice (*P* < 0.05).

Significant pulmonary lipid peroxidation was found after 48 hr exposure to 0.3 ppm O_3_ and 24 hr PE to 2 ppm O_3_ in *Nrf*2^+/+^ mice (Figure 4(a)). Compared to *Nrf*2^+/+^ mice, we found significantly greater and earlier lung lipid peroxidation in *Nrf*2^−/−^ mice during 0.3-ppm O_3_ (6 hr) while O_3_-induced lipid oxidation status was similar between two genotypes at other time points (Figure 4(a)). Acute O_3_ exposure caused significantly greater lung lipid peroxidation at 24 hr PE in *Nrf*2^−/−^ mice than in *Nrf*2^+/+^ mice (Figure 4(a)). The kinetics of lung lipid peroxidation and protein oxidation were not the same in the two O_3_ exposure models (Figures 4(a) and 4(b)). Mean protein carbonyl groups were greater in *Nrf*2^−/−^ mice than in *Nrf*2^+/+^ mice after air exposure (Figure 4(b)). The amount of protein carbonyl group was significantly increased over the air control after 3 d exposure to 0.3-ppm O_3_, and the O_3_-induced protein oxidation was significantly greater in *Nrf*2^−/−^ mice than in *Nrf*2^+/+^ mice after 2-3 d exposure. The effects of 2 ppm O_3_ on protein oxidation were found at 3 h PE, and no significant effect of genotype was found (Figure 4(b)). Different from lung tissue levels [29], no Nrf2-dependent glutathione depletion was found in ELF of air-exposed control mice (Figure 4(c)). Total glutathiones (oxidized GSSG and reduced GSH) in BAL fluids were significantly enhanced after 6 hr of 0.3-ppm O_3_ in both genotypes. Glutathione level in *Nrf*2^+/+^ mice remained elevated up to 72 hr of 0.3 ppm O_3_, while it significantly declined from 48 hr O_3_ in *Nrf*2^−/−^ mice (Figure 4(c)); this decline occurred simultaneously with increases in protein and lipid oxidations in these mice (Figures 4(a) and 4(b)). Acute exposure to O_3_ also significantly increased total BAL glutathione in *Nrf*2^+/+^ mice but not in *Nrf*2^−/−^ mice (Figure 4(c)).

### 3.5. Pulmonary Nrf2 and Antioxidant Activation

**Figure 5:**
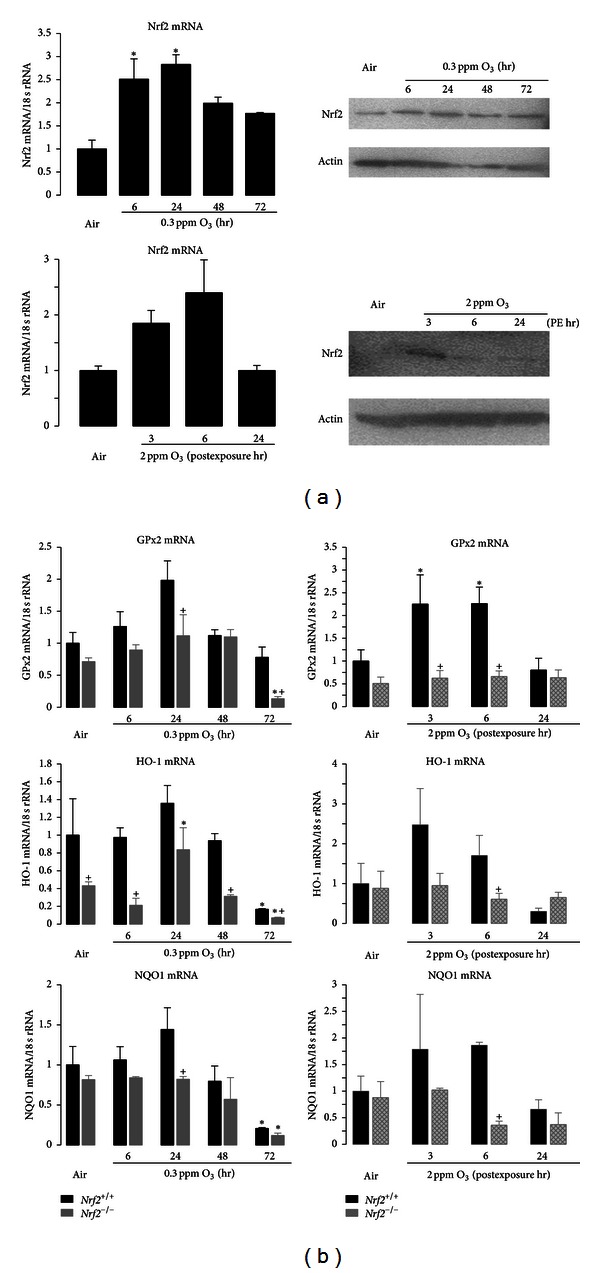
Lung Nrf2 and antioxidant expression. (a) O_3_-induced changes in expression of Nrf2 mRNA (left panels) and protein (right panels) in lung homogenates from *Nrf*2^+/+^ mice after exposure to air, 0.3 ppm O_3_ (top), or 2 ppm O_3_ (bottom). Data presented as mean ± SEM (*n* = 3-4/group) after normalization to air controls. * Significantly different from air control mice (*P* < 0.05). For Western blots, pan-actin was measured as a loading control. Representative band images from replicates are shown. (b) mRNA expression of antioxidants glutathione peroxidase 2 (GPx2), heme oxygenase-1 (HO-1), and NAD(P)H:quinone oxidoreductase 1 (NQO1) in lung homogenates from *Nrf*2^+/+^ mice after exposure to air, 0.3 ppm O_3_ (top), or 2 ppm O_3_ (bottom). Data present fold differences of each gene expression relative to *Nrf*2^+/+^ air after normalization to corresponding 18 s rRNA expression. Group mean ± SEM presented (*n* = 3-4/group). *Significantly different from genotype-matched air control (*P* < 0.05). ^+^Significantly different from exposure-matched *Nrf*2^+/+^ mice (*P* < 0.05).

Compared to air-exposed controls, mRNA expression of lung Nrf2 in *Nrf*2^+/+^ mice was significantly enhanced after 6 and 24 hr exposure to 0.3 ppm O_3_ and declined thereafter (Figure 5(a)). Lung protein level of Nrf2 remained elevated after 72 hr O_3_ (Figure 5(a)). Following acute exposure to 2 ppm O_3_, Nrf2 message level appeared to increase relative to air-exposed mice, but these increases were not statistically significant (Figure 5(a)). Relative to air control mice, lung Nrf2 proteins also increased 3 hr after exposure to 2 ppm O_3_ (Figure 5(a)). We also characterized expression profiles of pulmonary ARE-responsive genes GPx2, HO-1, and NQO1 after O_3_ exposure. The kinetics of message levels for the genes were largely similar to those of Nrf2 (Figure 5(b)), with increases after 6 and 24 hr exposure to 0.3 ppm O_3_ and increases at 3 and 6 hr PE to 2.0 ppm O_3_. Nrf2-dependent differences in mean gene expression levels were found after air exposure in HO-1, after exposure to 0.3 ppm O_3_ in GPx2 (48 and 72 hr), HO-1 (6, 24, and 48 hr), and NQO1 (24 hr), and after exposure to 2.0 ppm O_3_ in Gpx2 (3 and 6 hr PE), HO-1 (6 hr PE), and NQO1 (6 hr PE) (Figure 5(b)). 

## 4. Discussion

Among components of ambient pollutions, O_3_ is one of the most intensively studied oxidants. However, despite the extensive research on health effects of exposure to O_3_, mechanisms of differential susceptibility among exposed humans and animals remain unclear. In the present study we found that, relative to wild-type mice, mice with targeted deletion of the transcription factor Nrf2 had greater numbers of inflammatory cells and markers of oxidative stress and diminished antioxidant capacity following exposure to 0.3 or 2.0 ppm O_3_. These studies support the hypothesis that Nrf2 has an important role in protecting the lung against the inflammation and injury induced by exposure to O_3_ and may lead to means for preventing injury induced by inhaled oxidants.

High concentrations of O_3_ (≥2 ppm) are not encountered in the outdoor environment. However, short exposures to high concentrations have been used to predict a possible human exposure during vigorous exercise at a high O_3_ concentration of approximately 0.4 ppm [31]. Acute exposures also provide a reproducible tool to examine molecular and cellular events underlying acute lung injury caused by oxidant overload. Sub-acute exposure (up to 72 hr) to 0.3 ppm O_3_ represents a more environmentally relevant dosing regimen and also elicits airways inflammation though airways hyperreactivity is not a strong feature of this model. Based on National Ambient Air Quality Standards for ambient O_3_ (8 hr average 0.075 ppm; details in http://www.epa.gov/air/criteria.html) and results from dosimetry studies in which rodents require 4-5-fold higher doses of O_3_ than humans to create an equal deposition and pulmonary inflammatory response [31], either level of O_3_ used in the current study is a reasonable exposure level which is comparable with humans exposures. Interestingly, some of the protective effects of Nrf2 were specific to the two exposure regimens. For example, significantly greater number of total cells and neutrophils were found in *Nrf*2^−/−^ mice relative to *Nrf*2^+/+^ mice after acute exposure to 2 ppm O_3_, while no genotype effects were found after exposure to 0.3 ppm O_3_. One reason for this difference may be attributed to a difference in the magnitude of the injury induced by two concentrations of O_3_ in the current models. The acute exposure model elicited a larger cellular inflammatory response (e.g., 20 × 10^3^ versus 2 × 10^3^ neutrophils), and it is possible that the protective effect of Nrf2 may not be manifested until greater injury and subsequent sequelae initiate Nrf2 activation. Conversely, loss of *Nrf2* caused increased BAL protein, epithelial cell loss, histopathological changes, and Muc5AC production in both models. The different protective effects of Nrf2 in the two models illustrate the complexity of the pulmonary response to oxidant stimuli and suggest that Nrf2 may have different protective capacities against environmental stressors that are dose-dependent.

A role for Nrf2 in response to other air pollutants has also been demonstrated. Particulate matter (PM) is known to be proinflammatory and generates ROS in airway cells and tissues, and studies have suggested a role for the Nrf2-ARE pathway in pulmonary defense against ambient PM exposures. For example, diesel exhaust particles (DEP) increased Nrf2 levels and ARE responses in airway epithelial cells [32]. *Nrf2*-deficient mice were significantly more susceptible to lung DNA adduct formation and allergic airway inflammation induced by DEP, compared to similarly exposed wild-type mice [33, 34]. Chronic exposure to nanosized PM also enhanced Nrf2 and ARE-responsive detoxifying enzymes in the lung [35]. Williams et al. [36] demonstrated that dendritic cells from *Nrf*2^−/−^ mice heightened Th2-type allergic responses including expression of surface antigens and production of interleukins 10 and 12 against ambient PM, compared to dendritic cells derived from wild-type mice. Supporting a role for Nrf2 in inflammatory allergic responses against airborne particles, polymorphisms in* NRF2* and ARE-responsive antioxidant genes (*GSTP1*, *SOD2*) were associated with a trend toward increased risk of hospitalization during periods of high outdoor PM in an asthma/COPD cohort [37]. In extra pulmonary tissues, potential protective roles of Nrf2-ARE in particulate toxicity have been addressed using mouse models of atherosclerosis [38], insulin resistance, and risk of type 2 diabetes [39].

Both O_3_ exposure regimens diminished total glutathione and increased markers of oxidant stress (oxidized proteins and lung lipid peroxidation) in the BAL fluid from *Nrf*2^+/+^ and *Nrf*2^−/−^ mice. In general, these effects were greater in *Nrf*2^−/−^ mice than in *Nrf*2^+/+^ mice. These results are consistent with the hypothesis that absence of *Nrf2* suppresses antioxidant capacity and leads to greater O_3_-induced production of oxidized molecules which contributes to enhanced inflammatory response in *Nrf*2^−/−^ mice compared to *Nrf*2^+/+^ mice. Although health effects of environmental O_3_ have been broadly examined (e.g., http://www.epa.gov/apti/ozonehealth/population.html), biochemical aspects of inhaled O_3_ and cellular and molecular mechanisms underlying pulmonary O_3_ toxicity are not fully understood. Due to limited water solubility, most of the inhaled O_3_ is known to reach the lower respiratory tract. O_3_ in the lung dissolves in the thin layer of ELF of the conducting airways, and reacts rapidly with various biomolecules, particularly those containing thiol or amine groups or unsaturated carbon-carbon bonds, and this reaction is thought to be mediated by ROS in the ELF. O_3_ itself or its reaction products (e.g., lipid ozonation products) react with underlying epithelial cells, immune cells, or neural receptors in the airway wall, and it may propagate inflammatory and allergic responses [40]. O_3_ also causes oxidative DNA fragmentation and adduct (8-oxo-dG) formation [41], which could involve the weak carcinogenic response in mouse lung after chronic exposure [5, 42]. Antioxidants in cells and the lining fluid are thought to protect the epithelial barrier against O_3_ or its reaction products. Therefore potentially important mechanisms contributing to respiratory pathogenesis of O_3_ include the imbalance between ROS and antioxidant capacity, and Nrf2 may have an important role in maintaining the balance. 

Results of our investigation lead to the possibility that dietary supplementation with antioxidants may prevent or suppress the toxic effects of exposure to O_3_. However, the effectiveness of antioxidant supplements (e.g., vitamins A, C, and E, N-acetylcysteine) remains inconclusive in human studies of O_3_ exposure [43]. In laboratory rodents, supplementation with gamma-tocopherol significantly attenuated allergic responses and mucus production in upper airways [44]. Servais et al. [45] found that immature (3 wk old) rats were more sensitive to O_3_ (0.5 ppm, 12 hr/d, and 7 d) in body weight loss and DNA adduct formation than adult (6 wk old) rats, and they attributed this difference to relatively lower SOD, GPx, and catalase in the immature rats compared to the adults. Moreover, mice overexpressing Cu/Zn SOD (SOD1) were also resistant to acute O_3_ (0.8 ppm, 3 hr)-induced edema, inflammation, and lipid peroxidation in the lung [46]. Recent studies demonstrated that ambient level of O_3_ increases Nrf2 and ARE responses in airway cells or in the lung [22–24], though little attention has focused on the role of Nrf2. In addition, mice genetically deficient in phase 2 detoxifying enzymes, direct Nrf2 effectors, have variable responses to O_3_. Enhanced inflammation, vascular permeability, and DNA adduct formation were found in the lung of metallothionein (*Mt1*/*Mt2*) null mice after sub-acute O_3_ (0.3 ppm, 65 hr) exposure [47]. In contrast, with 70% depletion of glutathione, reduced lung injury was found in mice deficient in modifier subunit of glutamate cystein ligase (*Gclm*) relative to their wild-type controls [48]. The authors suggested that compensatory magnification of antioxidant defenses such as metallothioneins, alpha-tocopherol transporter protein, and solute carrier family 23 member 2 (sodium-dependent vitamin C transporter) in *Gclm* ^−/−^ mice may confer increased resistance to O_3_-induced lung injury [48]. Similarly, mice genetically deficient in peroxiredoxin (*Prdx1*) were more protected against acute O_3_ (2 ppm, 6 hr)-induced lung inflammation compared to wild-type mice, and Prx1 as a potent pro-inflammatory factor activating toll-like receptor 4/NF-*κ*B signaling was thought to recruit the inflammatory regulators in the model [22]. Overall, deletion of single defense enzyme may not be sufficient to affect airway pathogenesis by acute or sub-acute O_3_. The protective effect of Nrf2 in O_3_-exposed lung was noticeable in anti-inflammation and redox balance as well as protection of airway cell death and exfoliation and mucus overproduction in either or both exposure periods. Inasmuch as emerging evidence indicates that Nrf2 not only modulates antioxidant enzymes but also affects various pathways including cell cycle and immunity directly through ARE target genes or indirectly through interaction with other signaling networks [26, 49, 50], Nrf2 may exert its defensive effect against O_3_ not only through antioxidant defense but also through mechanisms such as activation of macrophage scavenger receptor [51] or inhibition of the inflammasome pathway [52]. 

Acute exposure to 2 ppm O_3_ did not alter airways reactivity in wild-type mice, and any effect of *Nrf2* deficiency on airway hyperreactivity in response to O_3_ could not be evaluated in the current study. It has been noted that changes in airways reactivity and inflammation/injury in response to O_3_ are not always codependent in rodents [53] or in human subjects [54, 55]. Furthermore, airways reactivity to acetylcholine is strain dependent [53]. The background strain (ICR) of the current study may have contributed to the low acetylcholine reactivity basally and after O_3_ exposure, considering that ICR mice are more like Th1-responders as they lack pulmonary eosinophilia and serum IgE induction after airway viral infection [29], compared to Th2-responder strains such as BALB/cJ. Alternatively, as severe mucus overproduction and hyper-secretion are the key phenotypes in the O_3_-susceptible *Nrf*2^−/−^ mice, it is also possible that airway plugging by excess mucus may hinder the access of aerosolized acetylcholine to the muscarinic receptors and interrupt the measurement of airway functions in these mice. Further investigations with targeted deletion of *Nrf2* on different strain backgrounds should provide insight to the role of Nrf2 on airway reactivity. 

## 5. Conclusion

 Genetic loss of *Nrf2* augmented pulmonary cellular toxicity including inflammatory cell influx, epithelial injury, and mucous cell hyperplasia leading to mucus hyper-secretion against ambient levels of O_3_. Heightened pulmonary oxidative stress indicated by lipid peroxidation after acute O_3_ exposure and protein oxidation after sub-acute O_3_ exposure parallel with suppressed antioxidant defense in *Nrf*2^−/−^ mice relative to their wild-type controls explain the protective role of Nrf2. Results suggest that therapeutic intervention of Nrf2 inducers for respiratory disorders may protect individuals at risk to environmental oxidants.

## Supplementary Material

Supplemental Figure 1 demonstrates airway responses to acetylcholine in *Nrf2^+/+^* and *Nrf2^−/−^* mice exposed to acute O_3_ at 24 hr PE. Total airway resistance (R, cmH_2_O*∙*s/mL) was measured in tracheotomized mice in response to increasing aerosolized acetylcholine concentrations (6.25-25 mg/mL) using the FlexiVent system. Airway reactivity was expressed as area under the curve (AUC, cmH_2_O*∙*s/mL x mg/mL) for R.

## Abbreviations

AB/PAS: Alcan blue/periodic acid Schiff
ANOVA: Analysis of variance
ARE: Antioxidant response element
AUC: Area under the curve
BAL: Bronchoalveolar lavage
BSA: Bovine serum albumin
DEPs: Diesel exhaust particles
DNP: 2,4-Dinitrophenylhydrazine
EKG: Electrocardiogram
ELF: Epithelial lining fluid
ELISA: Enzyme-linked immunosorbent Assay
*Gclm*: Glutamate cysteine ligase, modulatory subunit (murine, gene)
GSH: Glutathione, reduced
GSSG: Glutathione, oxidized
GST: Glutathione S-transferase
GPx: Glutathione peroxidase
HBSS: Hank's balanced salt solution
H&E: Hematoxylin and eosin
HO-1: Heme oxygenase-1
HRP: Horseradish peroxidase
IgE: Immunoglobulin E
LDH: Lactate dehydrogenase
MDA: Malondialdehyde
MT: Metallothionein
Muc5AC: Mucin 5, subtypes A and C
NADPH: Nicotinamide adenine dinucleotide phosphate, reduced
NIEHS: National Institute of Environmental Health Sciences
NQO1: NAD(P)H:quinone oxidoreductase 1
Nrf2,Nfe2l2: NF-E2 related factor 2
O_3_: Ozone
PE: Postexposure
PMs: Particulate matters
ppm: Parts per million
Prdx1: Peroxiredoxin 1
RIPA: Radioimmunoprecipitation assay
ROS: Reactive oxygen species
RT-PCR: Reverse transcription-polymerase chain reaction
SDS-PAGE: Sodium dodecyl sulfate-polyacrylamide gel electrophoresis
SOD: Superoxide dismutase
TBA: Thiobarbituric acid
Th: T helper cell immune response
TMB: 3,3′5,5′-Tetramethylbenzidine
TNF-*α*: Tumor necrosis factor alpha.
