# Supplementary material for: Exacerbated Airway Toxicity of Environmental Oxidant Ozone in Mice Deficient in Nrf2
Source: Oxid Med Cell Longev. 2013 May 9;2013:254069. doi: 10.1155/2013/254069 (PMC3665255; doi:10.1155/2013/254069)
Supplement: Supplementary file 1 — Supplemental Figure 1 demonstrates airway responses to acetylcholine in Nrf2+/+ and Nrf2−/− mice exposed to acute O3 at 24 hr PE. Total airway resistance (R, cmH2O∙s/mL) was measured in tracheotomized mice in response to increasing aerosolized acetylcholine concentrations (6.25-25 mg/mL) using the FlexiVent system. Airway reactivity was expressed as area under the curve (AUC, cmH2O∙s/mL x mg/mL) for R. [file 254069.f1.pdf]

Supplementary Material

Supplementary Figure 1 demonstrates airway responses to acetylcholine in *Nrf2*$^{+/+}$ and *Nrf2*$^{-/-}$ mice exposed to acute O_3 at 24 hr PE. Total airway resistance (R, $cmH_2O{\cdot}s/mL$) was measured in tracheotomized mice in response to increasing aerosolized acetylcholine concentrations (6.25-25 mg/mL) using the FlexiVent system. Airway reactivity was expressed as area under the curve (AUC, $cmH_2O{\cdot}s/mL$ x mg/mL) for R.

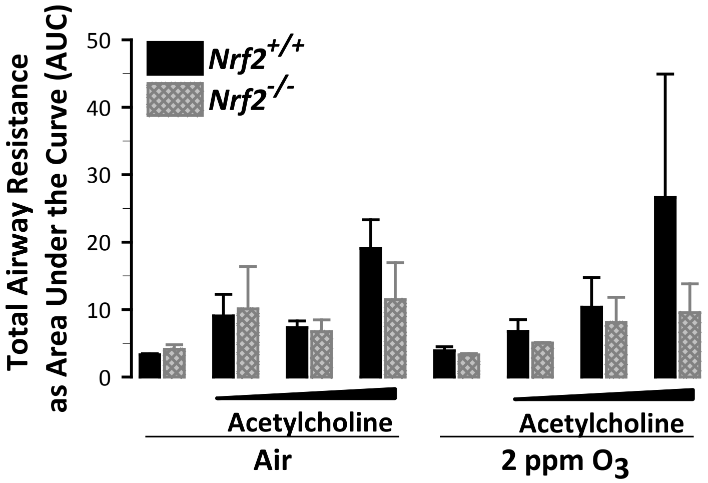

Supplementary Figure 1. Airway responses to acetylcholine. Response to acetylcholine (6.25-25 mg/mL) was determined using the FlexiVent system in tracheotomized mice exposed to air or 2-ppm O_3 (24 hr post-exposure). Total airway resistance (R, $cmH_2O{\cdot}s/mL$) measured by the FlexiVent was expressed as area under the curve (AUC, $cmH_2O{\cdot}s/mL$ x mg/mL) for resistance (R) after increasing acetylcholine concentrations.
